# A survey of path planning of industrial robots based on rapidly exploring random trees

**DOI:** 10.3389/fnbot.2023.1268447

**Published:** 2023-11-03

**Authors:** Sha Luo, Mingyue Zhang, Yongbo Zhuang, Cheng Ma, Qingdang Li

**Affiliations:** College of Electromechanical Engineering, Qingdao University of Science and Technology, Shandong, China

**Keywords:** industrial robot, path planning, RRT algorithm, mobile robot localization, robot

## Abstract

Path planning is an essential part of robot intelligence. In this paper, we summarize the characteristics of path planning of industrial robots. And owing to the probabilistic completeness, we review the rapidly-exploring random tree (RRT) algorithm which is widely used in the path planning of industrial robots. Aiming at the shortcomings of the RRT algorithm, this paper investigates the RRT algorithm for path planning of industrial robots in order to improve its intelligence. Finally, the future development direction of the RRT algorithm for path planning of industrial robots is proposed. The study results have particularly guided significance for the development of the path planning of industrial robots and the applicability and practicability of the RRT algorithm.

## 1. Introduction

**Figure 1:**
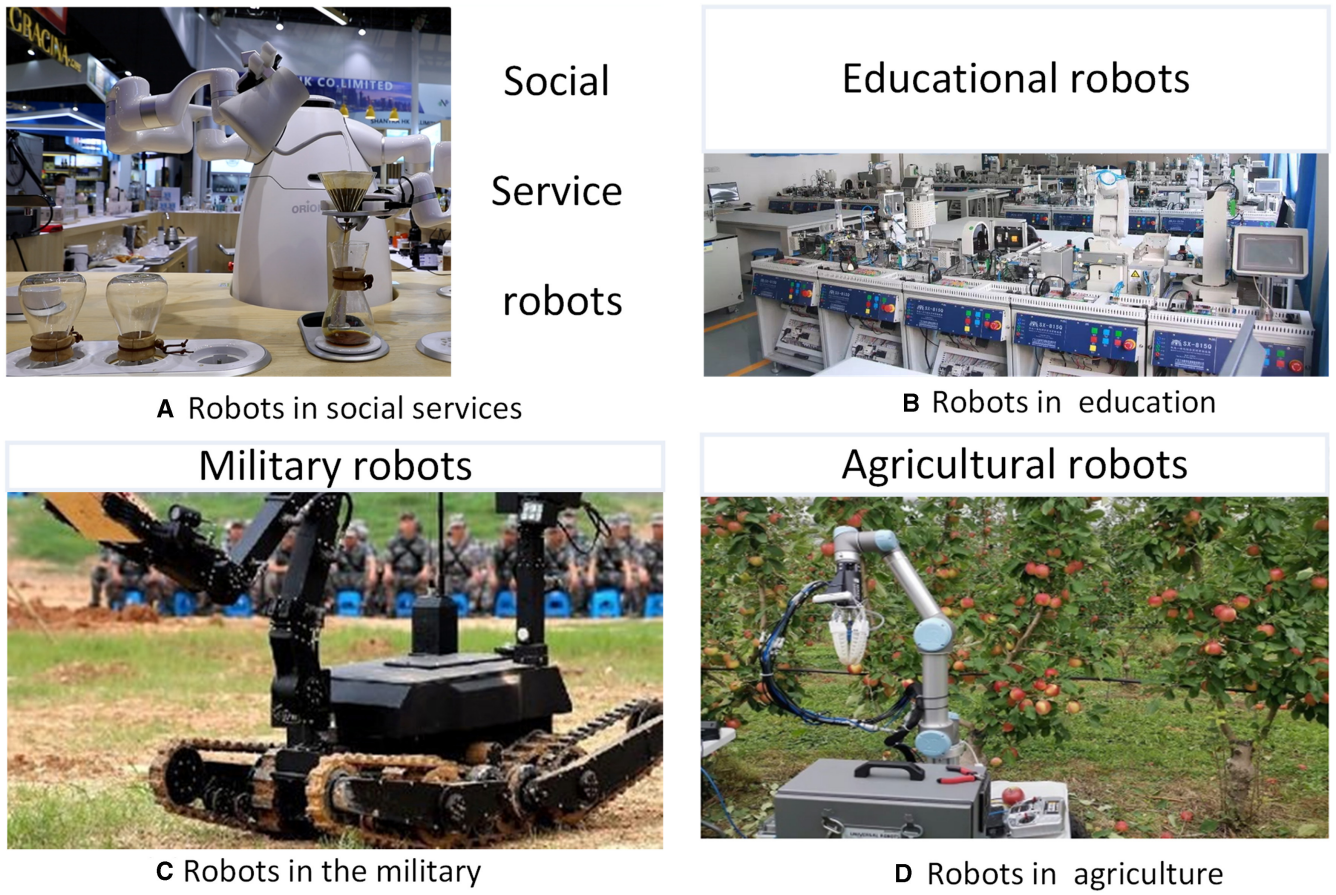
The application scenarios of robots (http://image.glgooo.top/). **(A**) Robots in social services. **(B)** Robots in education. **(C)** Robots in the military. **(D)** Robots in agriculture.

Robotics has demonstrated its power since the concept of robots was first proposed in the 1850s. With the development of information technology, communication technology, artificial intelligence, sensor technology, navigation technology, control technology, etc., robotics has been evolving for nearly 70 years, and it has become a high-tech technology integrating cybernetics, mechanisms, bionics, artificial intelligence, and other disciplines. Meanwhile, the structure and function of robots also tend to be diversified. In the context of the deep integration of artificial intelligence, the internet, big data, and cloud computing, the perception and decision-making cognitive ability of robots have been improved with the help of supercomputing ability. Also, the robot has stronger flexibility, versatility, environmental adaptability, and autonomy; it can adapt to more complex and changeable application scenarios. As an integrator of automation equipment, the application scope of robots covers all aspects of people's production and life from industrial manufacturing to social services, military, education and entertainment, agricultural production, etc. (shown in Figure 1). At present, there are many types of robots, and some scientists classify robots into industrial robots, service robots, and special robots (Jiawei, 2018). The industrial robot is a multi-joint manipulator, mechanical arm, or multi-degree of freedom robot for the industrial sector. Service robots mainly refer to human services, e.g., maintenance, security, rescue, guardianship, etc. Besides, special robots are advanced robots that are suitable for special application scenarios, special structures, and special functions, e.g., micro-nano robots, deep-sea robots, bionic robots, etc. In industry, jointed industrial robots are widely used in welding, spraying, handling, assembly, machining, and other fields (Patle et al., 2019).

In the context of the continuous development of the global robotics industry and the new generation of technological revolution, industrial robots have gradually become the core equipment of national industrial development. Also, they are known as the crown jewel of the high-end equipment manufacturing industry, which reflects a country's industrial development level and comprehensive strength. Therefore, robotics has been included in the 21^st^ century national high and new technology development plan by many countries around the world. In terms of global robot applications, the typical application scenarios of robots include automobile manufacturing, electronics manufacturing, storage and transportation, medical rehabilitation, emergency rescue, etc. With the help of the new generation of information technology, the applications of robots continue to expand. Meanwhile, according to the report released during the 2022 World Robot Conference, the global robotics market is expected to reach $51.3 billion in 2022, with an average annual growth rate of 14% from 2017 to 2022. Specifically, the market size of industrial robots, service robots, and special robots will reach $19.5 billion, $21.7 billion, and more than $10 billion, respectively. It is expected that by 2024, the global robot market will surpass $65 billion.

**Figure 2:**
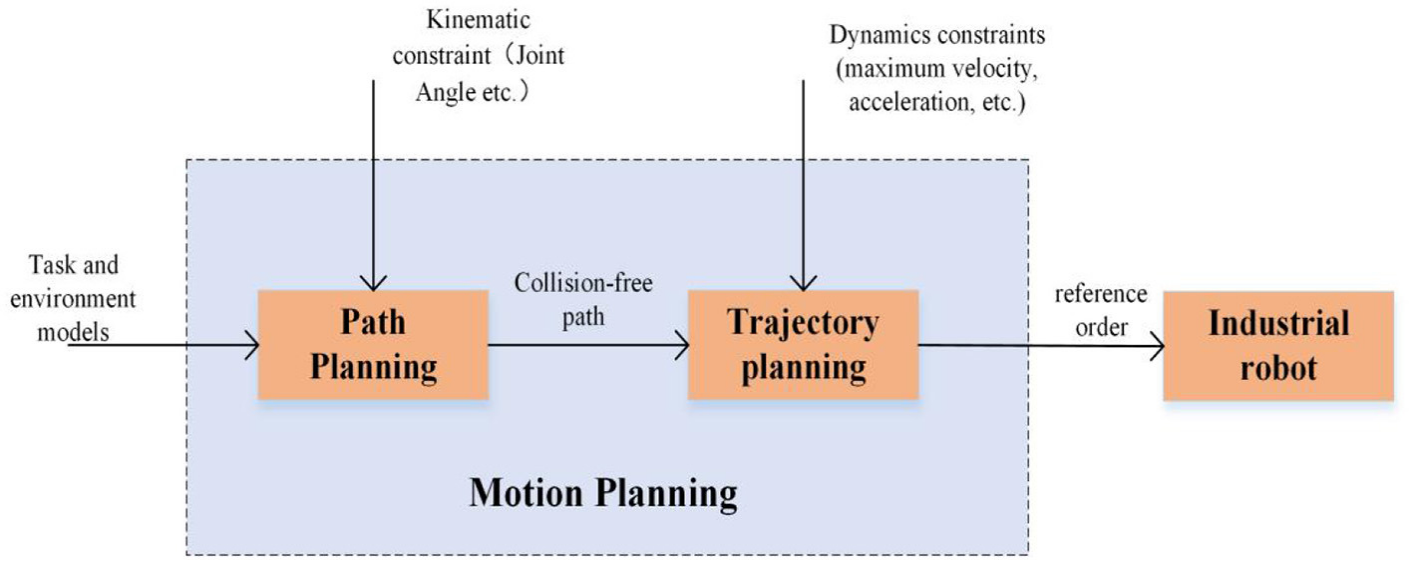
The role of robot path planning.

If the application of robotics is to develop at a high speed, robotics must first develop. With the development of robot technology and application, the path planning of robots as the foundation and support of robotics (shown in Figure 2) is one of the essential indicators of robot automation and intelligence. It has become one of the important branches in this research field. Meanwhile, path planning has been relevant to a range of tasks in industrial robots over the last three decades (Mithun et al., 2021). However, in the background of complex industrial robot applications, people put forward higher requirements for objective task control of industrial robots. In industry, path planning of industrial robots can enable them to complete the task while avoiding collision in a complicated environment. Especially, in the wave of the fourth industrial revolution, smart factories and intelligent manufacturing have developed rapidly. Multi-machine collaboration and human-machine integration whose core are industrial robots have become the key pillars of intelligent manufacturing (Balomenos et al., 2005). In order to ensure the safety of people and robots and complete the task efficiently, the dynamic path planning of industrial robots is particularly important. For the dynamic obstacles in the environment, the real-time obstacle avoidance path planning of industrial robots has become a difficult problem in international research.

Recently, the development of industrial robots is still in the first or second generation. The characteristics of industrial robots at this stage are that all tasks need manual debugging and teaching. This not only requires high labor and time costs but also affects the development of automation and intelligence of industrial robots (Alterovitz et al., 2016). Most importantly, the accuracy is low, the generalization ability is poor, and there are safety risks. Therefore, with the development and application of vision technology, sensing and recognition techniques are developing rapidly. This not only brings opportunities but also challenges for the intelligence of robots. From the perspective of opportunities, vision technology such as sensor and recognition technology can provide visual support for the intelligence of robots (Shi et al., 2023a,b). For challenges, path planning is in urgent need to match with the vision technology of industrial robots. Specifically, path planning of industrial robots uses vision technology to determine the pose of the target object and feedback to the industrial robots. Then, industrial robots plan the optimal path, mobilize the motion of each joint, and finally complete the corresponding task. The whole system with high precision, real-time, and intelligence represents the trend of industrial robot application in the future. From the perspective of the application object, path planning is a vital part of the industrial robot system. The high quality and efficiency of path planning enable industrial robots to complete their tasks safely, which is an important approach to save time and cost as well as reduce body wear. Many scholars have studied the path planning of industrial robots and made a lot of achievements. However, due to the complex process of path planning of industrial robots, there are still many immature aspects, e.g., the consideration of the planning dimension, the collision detection of industrial robots, etc. The path planning of industrial robots has important value and practical significance.

In order to make industrial robots more suitable for complex application scenarios, this paper comprehensively analyzes the particularity of path planning of industrial robots and then reviews the development of the RRT algorithm to promote the development of industrial robot path planning algorithm. The advantages of this paper are summarized as follows:

- From a new perspective, this paper analyzes the classification of path planning of robots, and summarizes the particularity of path planning of industrial robots.
- In terms of the overview of RRT algorithms, it innovatively takes four steps, which are sampling, measuring connection, collision detection, and path query. The development status and problems of the RRT algorithm are reviewed from these four aspects.
- Finally, the paper proposes the development trend of the RRT-based path-planning algorithm of industrial robots.

The rest of this paper is arranged as follows: In Section 2, the principle of path planning of robots is analyzed, in which the traditional path planning algorithms and the classification of path planning algorithm of robots are presented. The particularity of path planning of industrial robots is emphatically summarized. In Section 3, the development of the RRT algorithms is reviewed in the sampling phase, connection measurement, collision detection, and path query, which is beneficial for the path planning of industrial robots in a high-dimensional space. Section 4 is the summary and analysis. In Section 5, the development trend of the path-planning algorithm of industrial robots based on the RRT algorithm is analyzed. Section 6 is the conclusion.

## 2. The principle of robot path planning

Path planning is the core technology for robots to realize collision-free paths and complete tasks. So firstly, the basic principle of path planning of robots is analyzed, and then the classification of path planning algorithms of robots from a new comprehensive perspective is presented. Finally, the particularity of path-planning algorithms of industrial robots is summarized in order to select the appropriate path-planning algorithm.

### 2.1. The classification of path planning of robots

The purpose of path planning is to solve the path given the geometric constraints (such as obstacles and maps). Essentially, path planning is to determine a non-collision path between the initial point and the target point in the space without a time component (Cheng et al., 2023). Specifically, it may be to plan a collision-free shortest path from the initial position to the target position for mobile robots in a two-dimensional space (Xie et al., 2019) or to plan a safe and non-collision path with a relatively optimized path or search time for industrial robots. Jiang Xinsong, the father of Chinese robotics, synoptically defined path planning as the task of determining a non-collision path between the initial state (including position and attitude) and the target state (including position and attitude) according to a certain evaluation criterion in an environment with obstacles (Hong et al., 2022). Different distributions of obstacles in the environment directly affect the path, and the target location determination is provided by the higher-level task decomposition module.

**Figure 3:**
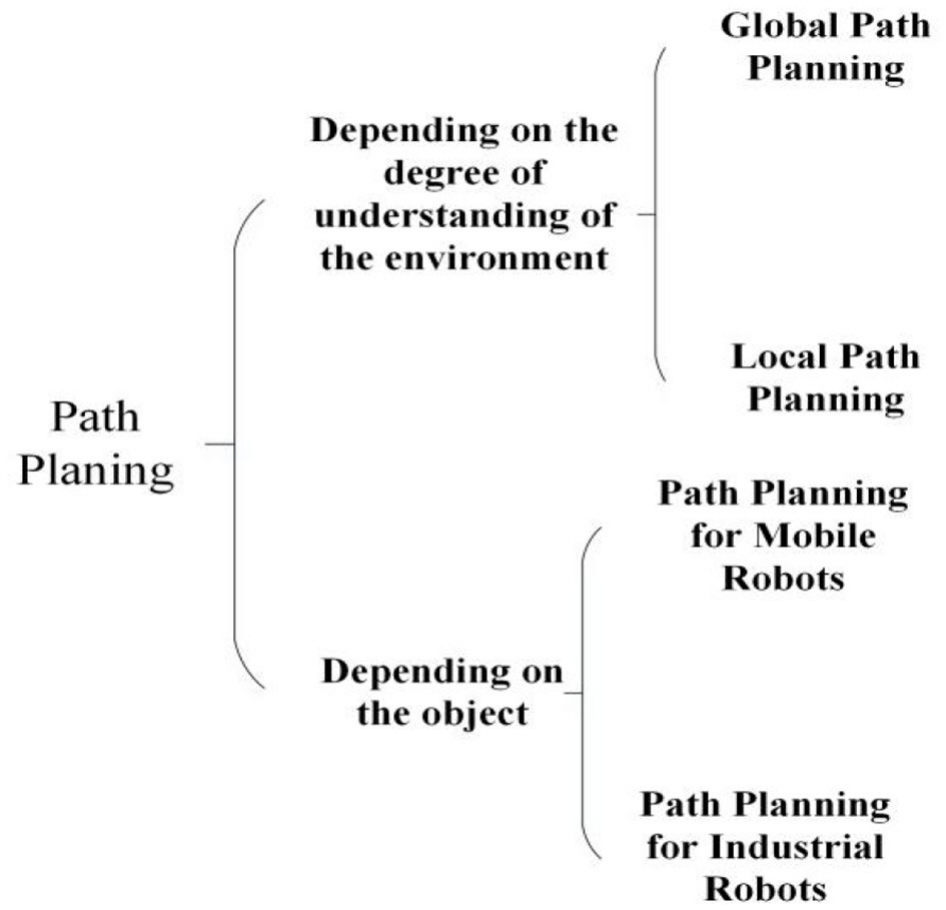
The classification of the path planning.

Path planning can be classified into different types according to different classification criteria, as shown in Figure 3. For instance, depending on the degree of environmental awareness, global path planning and local path planning are two main forms of path planning. Global planning is to fully understand the environmental information, while local path planning only needs to understand the obstacle information around the robot. From the perspective of obstacle information, global path planning is a static process, and local path planning is a dynamic process. According to different objects, path planning mainly serves mobile robots and industrial robots. The solution to path planning of mobile robots is to treat the mobile robot as a particle that can reduce the calculation amount in the planning process, especially in the collision detection process. In this process, the size problem caused by the robot's position transformation is solved by map expansion. In contrast, path planning of industrial robots needs to consider collision-free and cross-free restrictions (the industrial robot body does not have a cross-collision). Also, its scheme is to help industrial robots complete the path planning problem through kinematics transformation.

### 2.2. The difference between path planning of industrial robots and mobile robots

As shown in Table 1, there are differences between the path planning of industrial robots and mobile robots in the configuration space, the collision detection process, and the involvement of kinematics. In terms of configuration space, the configuration space of path planning of industrial robots is higher than in the non-European type space. Specifically, each joint of an industrial robot belongs to one dimension, so the spatial dimension of path planning of an industrial robot with more than three degrees of freedom is higher than three. It was proved that the configuration space of an industrial robot is a high-dimensional ring space. In terms of the complexity of collision detection, the collision detection process of the path planning of industrial robots should consider the collision detection of industrial robot actuators and realize the obstacle avoidance of each link of the robot arm (Szabó and Szádeczky-Kardoss, 2019). In terms of the involvement of kinematics, depending on different planning tasks, path planning of industrial robots can be divided into joint space (JS) path planning and Cartesian space (CS) path planning. The so-called JS path planning directly gives the expected path of the joint space, and this process needs to convert the end path points of the industrial robot into the corresponding joint Angle value according to the inverse kinematics equation. CS path planning describes the path sequence at the end of industrial robots in the task Cartesian space. In order to execute the path, it is also necessary to use the inverse kinematics equation of the industrial robot to convert the end-effector pose into joint value. Therefore, no matter the configuration space, path planning of industrial robots involves a kinematics solution.

**Table 1:** The difference in path planning between industrial robots and mobile robots.

**Types of path planning**	**Configuration space**	**Collision detection**	**The involvement of kinematics**
Mobile robot path planning	Two or three dimensions European space	Only considering the collision detection of robot actuators	NO
Industrial robot path planning	High-dimensional non-European space	Considering the collision detection of industrial robot actuator and realizing the obstacle avoidance of each link of the robot arm	YES

In summary, the complexity of path planning of industrial robots is higher than that of mobile robots. It involves more factors and processes and has higher requirements for planning algorithms. Therefore, the algorithm of path planning of mobile robots in two or three-dimensional space cannot be applied to industrial robots whose path planning is in high-dimensional non-European space. It is urgent to design a set of general path-planning algorithms for multi-degree-of-freedom industrial robots so that they can avoid obstacles and obtain the relatively optimal path in a complex environment.

The characteristics of path planning of industrial robots can be summarized as follows:

A) The path planning of industrial robots has a higher dimension;
B) The collision detection process in path planning of industrial robots is more complex;
C) The path planning of industrial robots involves kinematics.

Due to the complexity of path planning of industrial robots, the efficiency and applicability of the path planning algorithm of industrial robots are particularly important. This research can not only improve the performance of path-planning algorithms but also improve the intelligence of industrial robots. It can also promote the development of the robot industry.

## 3. Path planning algorithms of industrial robots

Based on the particularity of high dimension and high complexity of collision detection of path planning of industrial robots, the paper analyzes the advantages and disadvantages of traditional path planning algorithms for industrial robots. Then, it innovatively divides the RRT algorithm into four steps, namely, sampling, measuring connection, collision detection, and path query, and reviews the RRT algorithm from these four aspects. Particularly, the RRT algorithm summarized in this paper is not limited to industrial robots in order to fully understand the development of the RRT algorithm.

### 3.1. Path planning algorithms of industrial robots

According to the principle of path planning, path planning can make industrial robots independently plan a continuous non-collision smooth path in the configuration space when they move in an environment with static and dynamic obstacles. In the process, various constraints such as environmental constraints, time constraints, and dynamic constraints of industrial robots must be satisfied. Since the industrial robot is a highly non-linear, multi-input, and multi-output complex coupling system with a high degree of freedom, the complexity of path planning of industrial robots increases exponentially with the degree of freedom of robots and in polynomial with the complexity of obstacles in the circumstances. Recently, the research on industrial robots mainly focuses on static path planning algorithms. In static planning, the CAD model of industrial robots and obstacles in the environment should be determined first, which makes it impossible for robots to realize dynamic obstacle avoidance in real-time. Mobile robots are equipped with external sensing devices such as liDAR and navigation maps; they can flexibly deal with obstacle avoidance problems in complex dynamic environments, but they are limited to two-dimensional planar motions. To sum up, various obstacle avoidance algorithms of mobile robots are not suitable for the path planning of industrial robots with high-latitude obstacle avoidance (Zhao et al., 2020). After years of efforts, the following three types of methods have been proposed for the path planning of industrial robots (as shown in Table 2).

**Table 2:** Path planning methods for industrial robots.

**Path planning methods**	**Representative algorithms**	**Advantages**	**Disadvantages**
Traditional obstacle avoidance planning methods	APF (artificial potential field method) (Khatib, 1986), A^*^ (Hart et al., 1968)	The principle is simple and easy to implement	The methods need a large amount of calculation, are easy to fall into local minimum value, and cannot be applied to higher-dimensional space path planning
Intelligent obstacle avoidance planning methods	Artificial neural network (Wang et al., 2009), ant colonies algorithm (Guan-Zheng et al., 2007)	Easy to implement without modeling the environment	The methods have randomness, the solution is not unique, and they are not suitable for high-dimensional spatial path planning
Obstacle avoidance planning method based on random sampling	PRM (Probalistic Roadmaps) (Kavraki et al., 1996), RRT (Rapidly-exploring Random Trees) (LaValle, 1998)	It does not depend on the robot's state space and is suitable for high-dimensional space path planning	The methods have randomness and a slow search speed

The symbol * represents a special flag for an improved RRT algorithm, which is a specific sign.

The traditional obstacle avoidance path planning algorithm has a simple structure, but it depends on the accuracy of the obstacle expression in the environment (Karaman and Frazzoli, 2011). Moreover, constructing the environment graph or traversing the global nodes also makes the algorithm suffer from more complex calculations and low efficiency (Latombe, 1999; Huang and Teo, 2019). Traditional obstacle avoidance planning algorithms can be applied to 2-dimensional or 3-dimensional Euclidean space, and as to whether they are suitable for high-dimensional path planning of industrial robots, the paper (Tamaki et al., 2023) proved that by limiting the joint Angle of industrial robots to a certain range, the local surface of the ring space of industrial robots and N-dimensional Euclidean space can realize local differential homeomorphism. However, according to the concept of local differential homeomorphism, path planning of 6-DOF industrial is equivalent to a 6-dimensional surface, which means the algorithm that works well in mobile robot planning cannot be applied to path planning of industrial robots. For example, although the raster method is theoretically feasible in high dimensional space, the raster discretization and the mapping of obstacles in the high dimensional space will occupy a large amount of computing memory. Similarly, it is difficult for the APF method to establish potential field functions in a high-dimensional configuration space. Although the APF method is directly applied in the path planning of industrial robots (Palmieri and Scoccia, 2021), the method is incomplete and not optimal, so it cannot guarantee the success of the path planning of industrial robots.

Compared with the traditional path planning algorithm, the intelligent obstacle avoidance path planning algorithm has a powerful learning ability, which makes the algorithm more adaptable to the environment and alleviates the constraints of the environment on the algorithm. However, this type of algorithm has complex structures and many parameters, and the determination and optimization process of parameters makes the algorithm inefficient.

The above two kinds of algorithms are not fully applicable to path planning in high-dimensional configuration space. In addition, in terms of computational efficiency, it is better to create a graph across the free space directly than to accurately represent the free space where the robot works or adjust the algorithm parameters frequently (Sandakalum and Ang, 2022). The possibility of path planning in high-dimensional space was proved, which solved the problem of path planning in chain robots and maze-like environments. Sampling-based path planning algorithms do not depend on the expression of obstacles in the environment, and they realize path planning by constructing graph networks or searching random trees by sampling points. Although these algorithms may take up a lot of computing resources in the search process, they can always obtain a non-collision path in a long enough time because the algorithms are not constrained by the environment. Another advantage of these algorithms is their good applicability to high-dimensional spatial path planning. Among the sampling-based path planning algorithms, PRM and RRT are the two most popular ones. However, due to the dependence on the geometry of obstacles in the environment, the PRM algorithm is only applicable to static path planning (Noreen et al., 2018), and the biggest defect of the algorithm is the limitation in narrow space and unnecessary collision detection (Katiyar and Dutta, 2022). The RRT algorithm is not only suitable for static environments in high-dimensional space but also dynamic environments and scenarios with varying constraints (Adiyatov and Varol, 2013). In contrast, the sampling-based RRT algorithm is more useful for high-dimensional non-Euclidean space of industrial robots in complex environments. Besides the path planning of manipulators (Weghe et al., 2007), the RRT algorithm is also applicable to space robots (Zhou et al., 2017), analog circuits (Ahmadyan et al., 2012), high-speed ultrasonic aircraft (Pharpatara et al., 2015), unmanned aircraft (Gan et al., 2009), unmanned ground vehicles (Lolla et al., 2014), etc. In order to obtain a more efficient and advanced RRT path-planning algorithm for industrial robots, this paper studies the RRT algorithms applied in various fields. On the aspect of environment, the paper (Du Toit and Burdick, 2011; Pfotzer et al., 2017) thought path planning in the dynamic environment was similar to that in the static environment. Specifically, path planning in the dynamic environment was considered as the modification that would be alternated with the planner and would update steps (Zucker et al., 2007). As the basic environment, this paper takes the static environment as the working environment regardless of which environment the reference algorithms are applied to.

The evaluation indexes of path planning algorithms include the probability completeness and optimality of the algorithm. In Sampling-based obstacle avoidance planning methods, the RRT algorithm has probability completeness but not optimality. Thus, scholars have investigated the RRT algorithm and its improved algorithms for years. In LaValle (1998) of Iowa State University proposed the rapid-exploring random trees (RRT) algorithm. The algorithm has been widely utilized by scholars because of its high efficiency in high-dimensional spatial path planning. The RRT algorithm has probabilistic completeness, indicating that this algorithm can always obtain a satisfying path in a long enough time (Zhang et al., 2022). However, this “randomness” makes the RRT algorithm blind to a certain extent, which also leads to its low efficiency. For the above defects, Professor Lavalle and Professor Kuffner proposed the bidirectional RRT algorithm in 2000 (LaValle et al., 2001). This algorithm adds an intermediate point between the initial and target position, and it builds and extends two random trees in parallel, which enables the algorithm to achieve better efficiency. Then, Sertac and Emilio (Karaman and Frazzoli, 2011) proposed the RRT^*^ algorithm to overcome the non-optimality of the traditional RRT algorithm in 2010, and the algorithm uses the cost function to optimize the selection of the parent node. In this way, the algorithm can easily obtain the asymptotically optimal solution. In Gammell et al. (2014) proposed Informed-RRT^*^, which introduced a state subset to optimize the sampling space to obtain the optimal path.

**Figure 4:**
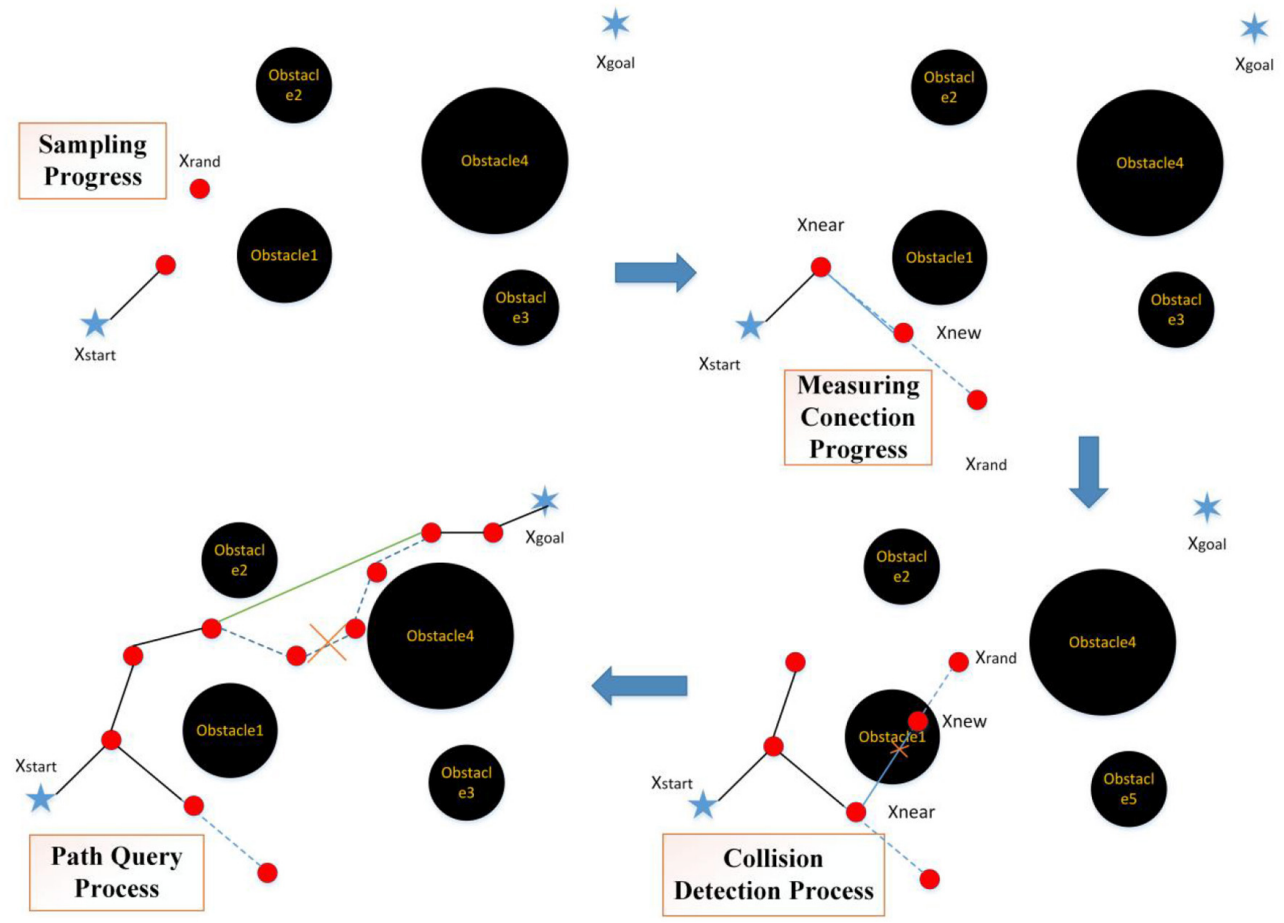
Implementation of the traditional RRT algorithm (*X*_start_, *X*_rand_, .., *X*_near_, and *X*_new_ represents the starting, random, end, near, and new points of path planning).

The above three algorithms are classic RRT algorithms, but neither of them fundamentally solves the randomness, inefficiency, and non-optimality of the algorithm. So, based on the above three algorithms, scholars further optimized the traditional RRT algorithm from four aspects (as shown in Figure 4): sampling progress, measuring connection process, collision detection, and path query.

**Figure 5:**
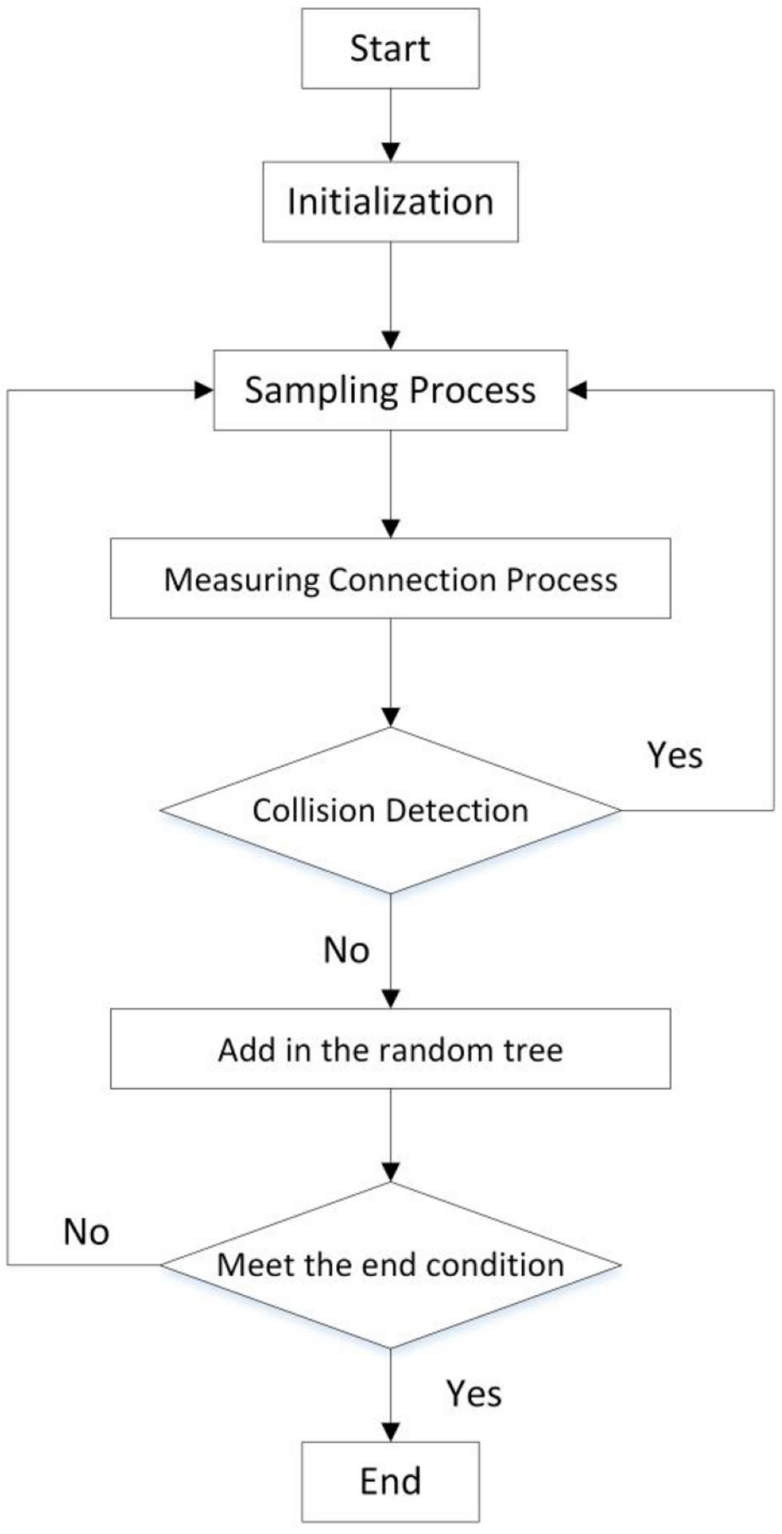
The flow chart of the traditional RRT algorithm.

The four stages of the traditional RRT algorithm are as follows (Elbanhawi and Simic, 2014) and its flow chart is shown in Figure 5:

A) Sampling progress: accessing all nodes in the robot configuration space with the same probability;
B) Measuring connection process: according to the constraints of the algorithm, selecting a node as a new node in the random tree;
C) Collision detection: detecting whether a collision occurs. If yes, the new node is discarded and resampling is performed; if no, the node is added to the random tree;
D) Path query: querying whether a node of the path is redundant. If yes, delete it; If no, keep it as the node in the random tree.

### 3.2. Sampling progress

Sampling is an important part of the RRT algorithm, and the sampling procedure usually samples in the uniform sampling mode by visiting all nodes in the space with the same probability (Véras et al., 2019). The uniform sampling method leads to low sampling efficiency and a large calculation amount of the algorithm, which also affects its efficiency and convergence. In terms of sampling strategy, scholars have improved the sampling process of the RRT algorithm in two directions: one is to change the probability of selection, and the other is to limit the sampling area.

**Figure 6:**
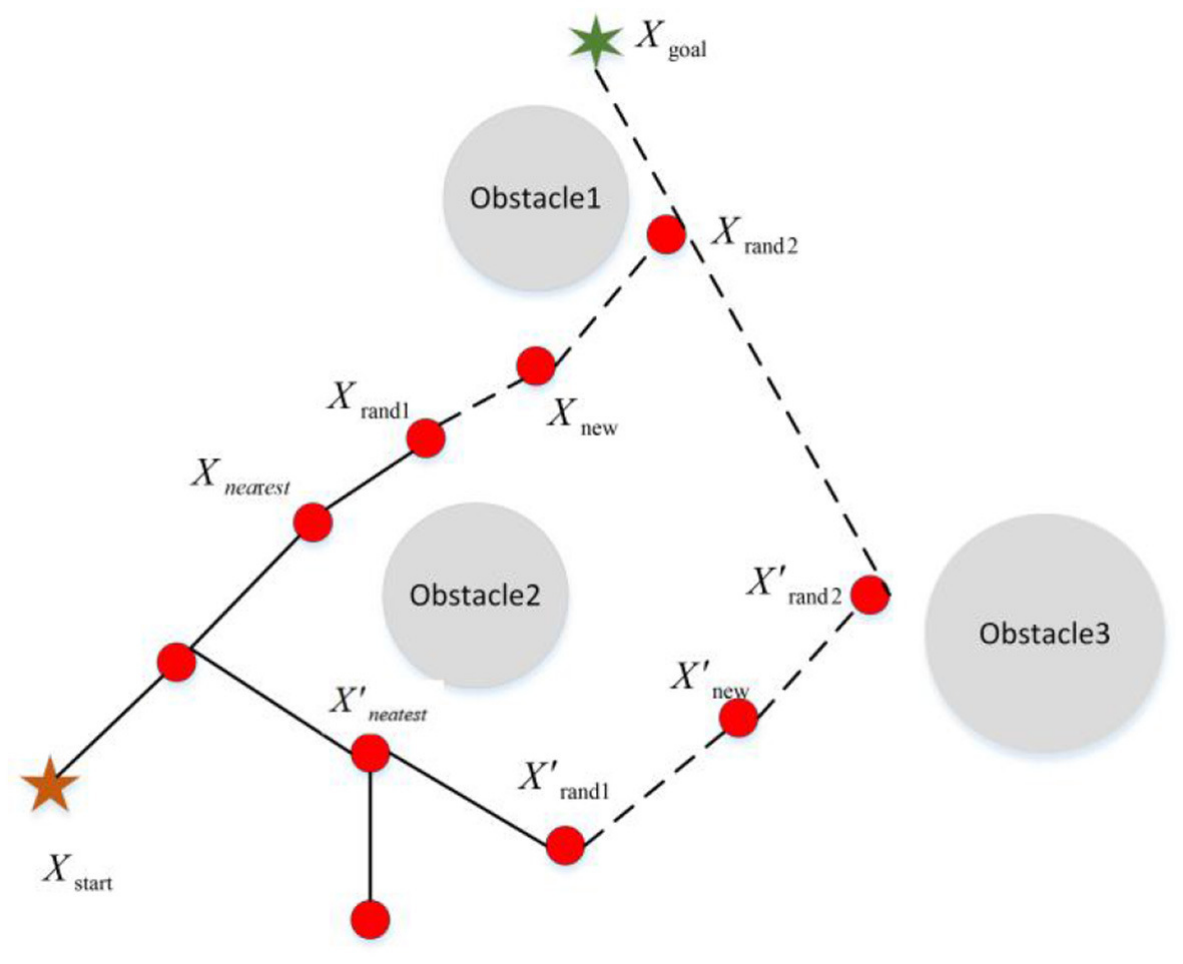
The schematic diagram of the target-biased strategy.

In terms of changing the probability of selection, Kang et al. (2016, 2019), Wei and Ren (2018), Huang and Teo (2019), Hu et al. (2020), Khan et al. (2020), Wang et al. (2021), and Wang et al. (2022a) introduced the target-biased method to realize “de-randomization” to a certain extent and achieve higher search efficiency. Although the target-biased method guides the sampling point to search toward the target point, there is no substantial change in terms of search space or the sampling point quality, and the redundant sampling of the RRT algorithm still exists (as shown in Figure 6).

*X*_start_ and *X*_goal_ are the starting and the end points of path planning. Path (*X*_start_-*X*_*nea*r*est*_-*X*_new_-*X*_rand2_) is the path generated by adopting the target-biased strategy. *X*_rand1_ and *X*^′^_rand1_ are random sampling points generated by the two strategies. *X*_*nea*r*est*_ and *X*^′^_*nearest*_ are the nodes closest to the random sampling points *X*_rand1_ and *X*^′^_rand1_ in the random tree of the two strategies. *X*_new_ and *X*^′^_new_ represent new nodes generated by different sampling strategies. *X*_rand2_ and respectively are the new random sampling points obtained by sampling the new node. It can be seen that the distance between the random sampling point *X*_rand2_ obtained by the target-biased and the target point is smaller. Although the target-biased strategy makes the sampling process of the RRT algorithm have a certain orientation, its search space is still large, and the phenomenon of redundant sampling still exists. Aiming at the sample space problem and drawing on the target-biased method, Liu et al. (2020) generated a search space for the RRT algorithm based on variable probability and obstacle density to make the search more efficient.

From the aspect of limiting the sampling area, Chi et al. (2021) proposed a heuristic sampling strategy for the RRT algorithm. The characteristic area was used as the sampling area, which helped to reduce the sampling range and obtain better search efficiency. Wang et al. (2020a) used the method of constantly changing the sampling area to guide the random tree expansion so that the algorithm can better adapt to the narrow region. For the quality of sampling points, Qureshi et al. (2013) and Xinyu et al. (2019) introduced the APF method into the RRT algorithm to choose high-quality sampling points. The APF method reduced redundant samples of the algorithm with the assistance of the attraction and the repulsion from the target point and obstacles. For the problem of low efficiency of the RRT algorithm, Qureshi et al. (2014) introduced the triangular geometry method to determine the sampling point through the centroid and interior of the triangle, which is also an extension of the strategy of reducing the search space. Lonklang and Botzheim (2022) minimized the number of unavailable nodes to generate a new sampling environment by reducing the obstacle area.

Based on the analysis of the sampling process of two commonly used improvement methods, Ganesan et al. (2021) proposed the G-RRT^*^ algorithm to address the slow convergence speed of the RRT^*^ algorithm. The G-RRT^*^ algorithm combines the target-biased method with the method of limiting the sampling area, thus generating sampling points closer to the target point by the target-biased method. This method can not only limit the sampling area but also reduce the number of visiting nodes. Also, simulation results indicate that the number of nodes on the path and the convergence speed of the algorithm can be greatly improved. So, it can be seen that the size of the sampling area, the target orientation, and the quality of the sampling points are important factors for the RRT algorithm to obtain better sampling efficiency, redundant sampling, and whole efficiency.

To show the effectiveness of algorithms, this paper compares some RRT variants with the traditional RRT algorithm from the aspect of the number of sampling nodes and planning time (as shown in Table 3). The comparison results show that target-bias strategy and heuristic sampling strategy enables the RRT algorithm to achieve better efficiency, and particularly speaking, in order to keep the algorithm under the same condition and reflect the algorithm performance, the data in Table 3 are the percentages obtained by comparing with the traditional RRT algorithm.

**Table 3:** The efficiency comparison of some algorithms.

**Algorithms**	**Improvement strategy**	**Planning time**	**Reduction rate of sampling points**
S-RRT (Wei and Ren, 2018)	Target-Biased	80.32%	77.11%
Improved RRT (Xia et al., 2022)	Heuristic sampling strategy	97.87%	97.69%
Variable Step-RRT^*^ (Liu et al., 2020)	Target-Biased + variable probability + obstacle density	49.34%	40.52%

The symbol * represents a special flag for an improved RRT algorithm, which is a specific sign.

### 3.3. Measuring connection process

The measuring connection process of the RRT algorithm is an important step in determining how to expand the whole algorithm, and it is also an important factor affecting the overall efficiency of the algorithm. The measuring connection process involves the generation and addition conditions of new nodes, the judgment of the nearest node, and the path cost. Scholars have improved the RRT algorithm from the above aspects to achieve better search efficiency.

**Figure 7:**
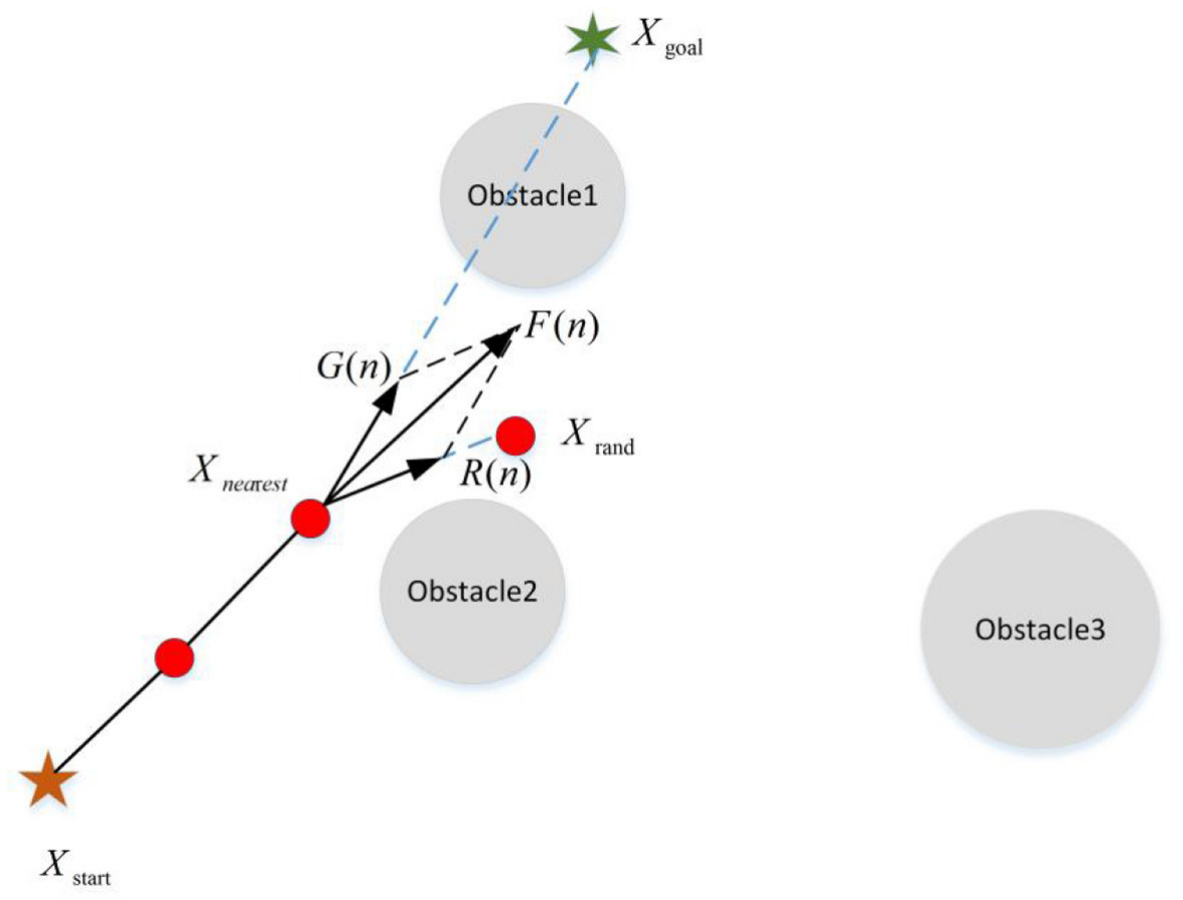
The strategy of adding the gravitational potential field. *G*(*n*) and *R*(*n*) represents the gravity from the target and the random sampling point, respectively. *F*_*att*_(*n*) is the resultant force of two gravitation forces whose direction indicates the direction in which the new node is generated.

**Figure 8:**
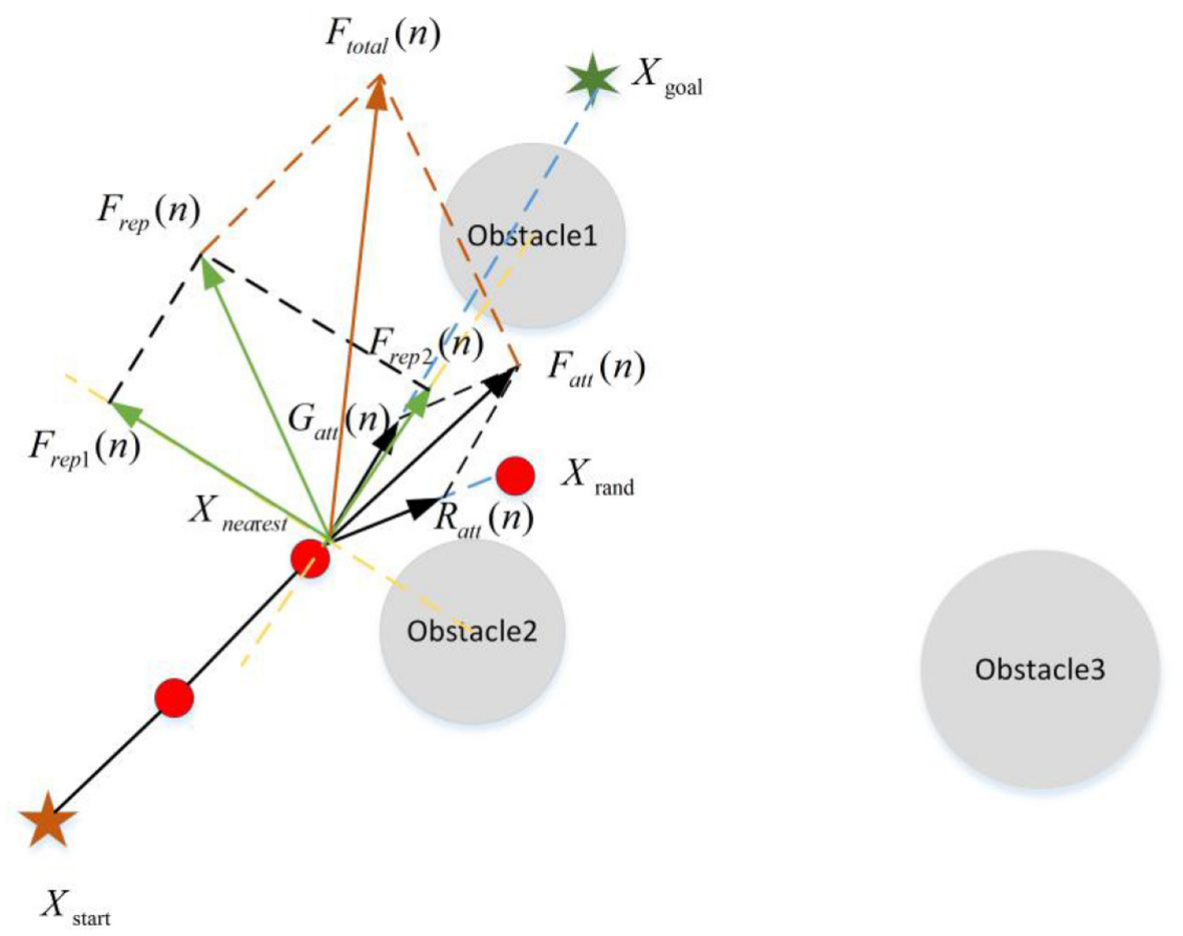
The strategy of adding the gravitational and repulsive potential field. *G*(*n*), *R*(*n*), and *F*_*att*_(*n*) are the same with the above Figure. *F*_*rep*1_(*n*) and *F*_*rep*1_(*n*) R respectively represent the repulsive force from two obstacles. is the result of two repulsive forces. *F*_*total*_(*n*) is the resultant force of attraction and repulsion whose direction indicates the direction in which the new node is generated.

For the generation of the new node, Qureshi and Ayaz (2016), Cao et al. (2019), Chen et al. (2021), Wang et al. (2021) and Yi et al. (2022) introduced the principle of APF into the RRT algorithm. With this method, the algorithm guides the production of new nodes by the gravitational effect of the target point (as shown in Figure 7). Meanwhile, the dynamic step strategy was adopted to make the improvement on the blindness and the search speed. However, in terms of APF, the gravitational potential field cannot fully describe the potential field action. Thus, Kabutan and Nishida (2018) and Wang et al. (2022a) not only introduced the gravitational potential field but also considered the influence of the repulsion of obstacles and realized the guidance effect of the real APF method on the new node (as shown in Figure 8). This method can improve the goal orientation and the ability of obstacle avoidance of the RRT algorithm. As shown in Figures 7, 8, the introduction of APF can provide theoretical guidance for the new node direction of the RRT algorithm, but the specific location of the new node cannot be determined. During the generation of new nodes, Adiyatov and Varol (2013) introduced the forced elimination strategy to obtain new nodes by considering factors such as the path cost of the node and whether there are child nodes. It makes the path quality greatly improved. In the new node extension, and Zhang et al. (2019), Liu et al. (2020), and Wang et al. (2020a, 2022b) introduced the dynamic step into the RRT algorithm to adaptively extend a certain step size to determine the location of new nodes. In this way, the RRT algorithm could ensure the success rate of new nodes in the area of dense obstacles and rapidly expand new nodes in the area of sparse obstacles, thus enabling the algorithm to achieve better efficiency.

In addition, as for the improvement in the asymptotic optimality of the path, in the measurement connection, Liu et al. (2020) and Wang et al. (2020b) took the path cost as the evaluation criterion to obtain the new node with the highest quality. Specifically, it requires the path cost of the new node to be a minimum in order to obtain the locally optimal path. Zhang et al. (2018) introduced a regression mechanism to select a new node with the smallest path for expansion, thus optimizing the local path. García et al. (2015) designed the path cost function by comprehensively considering the length and the average cost of the path, and the change of the cost with the path. Based on this, the RRT algorithm can obtain the node with the minimum path cost as the new node, thus improving the quality of the path. Besides, Szabó and Szádeczky-Kardoss (2019) introduced the transition test and minimum control strategy into the RRT algorithm. The path cost and the change of the new node were tested in real-time to determine whether the new node was available. According to the above analysis, the direction and quality of new nodes are the key factors of the RRT algorithm to achieve better efficiency in terms of measurement connection.

To show the effectiveness of the improved strategy in the measuring connection process, Table 4 presents the improvement of some improved RRT algorithms in planning time and length of the algorithm compared with the traditional RRT algorithm. Particularly, in order to keep the algorithm under the same condition and reflect the algorithm performance, the data in Table 4 are the percentages obtained by comparing with the traditional RRT algorithm.

**Table 4:** Comparison of indexes of the improved algorithms.

**Algorithms**	**Improvement strategy**	**Planning time**	**Percentage reduction of path length**
Variable Step-RRT^*^ (Liu et al., 2020)	Dynamic step size + path cost function designing	49.34%	9.18%
Improved RRT (Chen et al., 2021)	Artificial potential field method -Gravitational potential field	88.52%	13.12%
Improved P-RRT^*^ (Yi et al., 2022)	Artificial potential field method -Gravitational potential field	96.28%	24.63%
Improved RRT	Artificial potential field method	9.59%	3.78%
Improved RRT (Lixin et al., 2021)	Artificial potential field method	69.28%	15.62%
Improved RRT (Zhang et al., 2018)	Regression mechanism	36.36%	1.07%

The symbol * represents a special flag for an improved RRT algorithm, which is a specific sign.

Thus, the measuring connection process of the RRT algorithm is a key factor affecting the overall efficiency and the path progressive optimality. Thus, the improved RRT algorithm can obtain better efficiency by measuring the direction of the new node generated in the measuring connection process. Meanwhile, the design of the path cost function is conducive to obtaining the asymptotic optimal path. However, the efficiency and the asymptotic optimality of the RRT algorithm are two mutually restrictive factors. The realization of the asymptotic optimality will result in a large computation amount, while the improvement of efficiency may sacrifice the asymptotic optimality of the path. The indicators in Table 4 are sufficient to illustrate the relationship between efficiency and progressive optimality. However, the planning efficiency of each algorithm has been greatly improved by adopting the above strategy, but its path length does not match the efficiency optimization. The efficiency of the improved P-RRT ^*^ algorithm (Yi et al., 2022) improved by 96.28% compared with the traditional RRT algorithm, and its path length increased only by 24.63%. Therefore, the future development of the RRT algorithm should take into account efficiency and path optimality in the measuring connection process.

### 3.4. Collision detection process

As for the collision detection process, Zhang et al. (2018) improved the RRT algorithm by introducing collision probability and a collision evaluation function. With the method, both the number of collision detections and its efficiency can be optimized. Liu et al. (2019) introduced grid search and coarse collision detection for sampling points to simplify collision detection and improve efficiency. Szabó and Szádeczky-Kardoss (2019) approximated the robot and the obstacle as a convex polygon and realized a non-collision path using the RRT algorithm. Although the collision detection process simplifies the geometric representation of the robot and obstacles, the representation of a convex polygon is not suitable for all robots and obstacles, so the collision detection accuracy is poor. Furthermore, the quality of the path will be affected too. Collision detection is the key for the RRT algorithm to obtain a non-collision path, which requires a large amount of computation. Therefore, simplifying the collision detection process is the key for the RRT algorithm to reduce the computation amount, save storage space, and improve efficiency.

The above collision detection aims at static obstacles, but for robots, the environment is often complicated, and static obstacles, dynamic obstacles, or both are possible working scenarios. Jiang et al. (2022) used cylindrical and spherical bounding boxes to construct collision detection models to realize dynamic obstacle avoidance in complex environments. In the whole process, the RRT algorithm is the key technology to realize the obstacle avoidance path planning. When dynamic obstacles were encountered, the path was optimized by global path planning and local re-planning to achieve dynamic obstacle avoidance (Hong et al., 2022). Similarly, Deng et al. (2016) proposed a local re-planning algorithm-dynamic RRT algorithm to replace global planning, which preserved the original points as much as possible. So, this algorithm can reduce the planning time, improve efficiency, and realize dynamic obstacle avoidance. However, the above two dynamic obstacle avoidance strategies adopt local planning when they encounter dynamic obstacles, which essentially fail to predict dynamic obstacles dynamically and suffer from poor real-time performance. Fan et al. (2020) used the improved APF method for dynamic path planning, in which the direction and size of the obstacles' speed were introduced into the traditional APF to realize dynamic collision detection.

For path planning, the collision detection process is very complicated, and its accuracy and speed determine the efficiency of the algorithm. However, with the increasing complexity of working scenarios of industrial robots, dynamic collision detection will become an important feature of intelligent industrial robots. Therefore, considering robot application scenarios and algorithm development, the future research direction of the RRT algorithm will involve the adaptability of the environment and real-time collision detection.

### 3.5. Path query process

**Figure 9:**
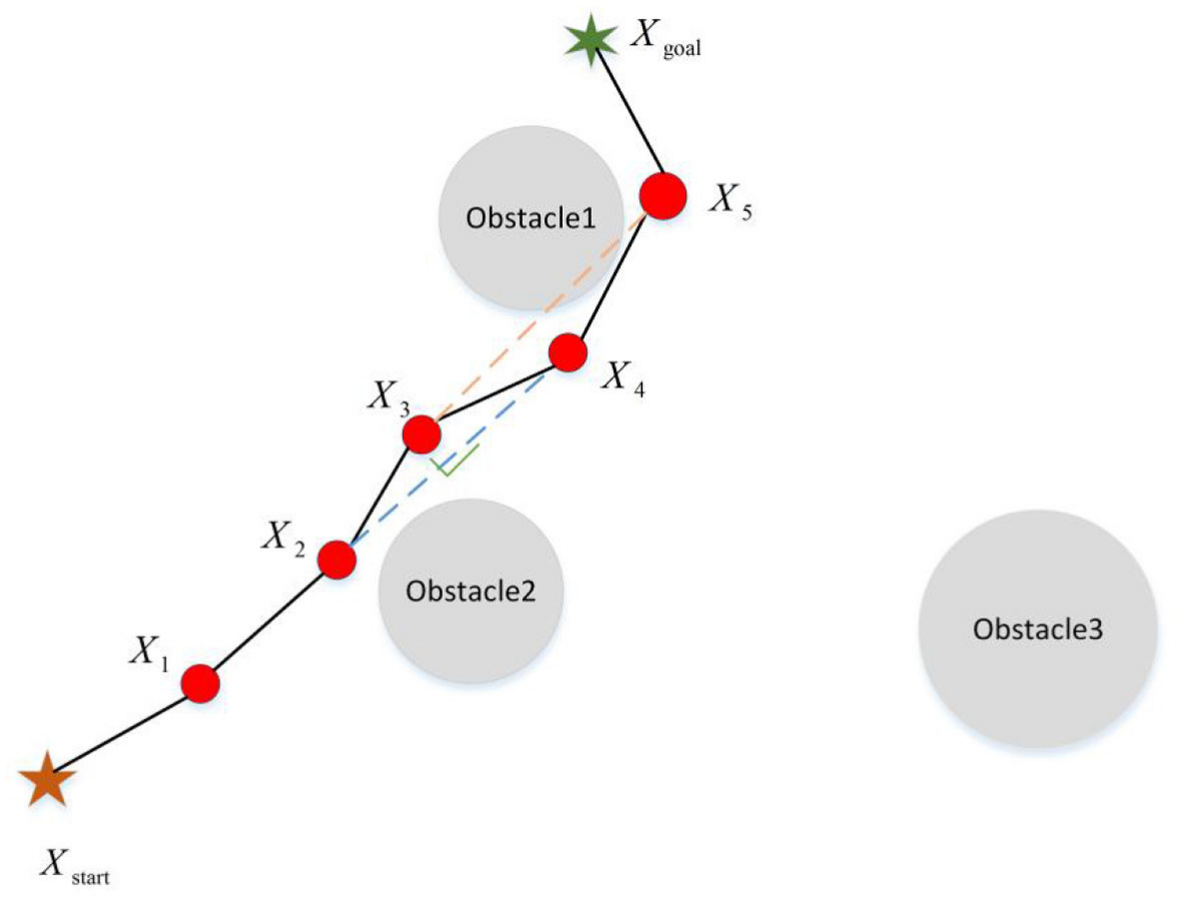
The schematic diagram of path query. After adopting the path query policy, *X*_3_ point can be deleted from Path (*X*_*start*_ − *X*_1_ − *X*_2_ − *X*_3_ − *X*_4_ − *X*_5_ − *X*_*goal*_), but *X*_4_ cannot be neglected because the line between point *X*_3_ and point *X*_5_ intersects the obstacle.

Path query is the core step to determine whether the RRT algorithm can achieve an asymptotic optimal or optimal solution (as shown in Figure 9). Li et al. (2018) designed a path reconstruction method that includes two steps: external extension and rewiring. This method optimizes the original path and eliminates redundant nodes to obtain the optimal path. Adiyatov and Varol (2013) optimized the path by limiting the number of nodes and eliminating the nodes without child nodes in the original path. Qureshi et al. (2014) combined the pruning strategy with limiting the number of nodes to eliminate redundant nodes and achieve the optimal path. Moreover, Jeong et al. (2019) considered the path cost of the parent node and ancestor node simultaneously. If the path cost is indeed reduced, this node is regarded as an effective node; otherwise, it is eliminated. This method enables the path to achieve asymptotic optimization. Cao et al. (2019) combined the RRT algorithm with the genetic algorithm, which introduced the path from the RRT algorithm as the initial condition into the genetic algorithm. Also, in this method, the fitness function is designed according to the path length and the distance between the robot arm and the obstacle to obtain the optimal path. At last, the redundant nodes are removed by the triangle inequality method to realize path optimization.

Currently, there are few studies on the path query process in the RRT algorithm, and the most used method is the pruning technique. However, in the application of the path planning algorithm, the optimization and the impact of the path on the robot (such as singular configuration and wear) are equally important for the path query process. Meanwhile, in terms of path optimality and practicability, the development direction of the RRT algorithm in the path query is to comprehensively consider the optimality of the algorithm and the constraints of robot kinematics and dynamics.

## 4. Summary and analysis

Aiming at the randomness, low efficiency, and non-optimality of the traditional RRT algorithm, methods such as target bias and APF have been introduced. However, the traditional RRT algorithm still has a low success rate and efficiency in narrow spaces and dynamic environments. Also, with the disadvantages of low efficiency and redundant sampling in the sampling process of the traditional RRT algorithm, the target-biased method improves its “randomness,” but the quality of the sampling points is the decisive factor of its sampling efficiency. Essentially, how to improve the quality of sampling points is the fundamental measure to improve the sampling efficiency and avoid redundant sampling.

The APF method enables the RRT algorithm to be goal-oriented for the problems of non-directivity and low expansion efficiency in the measuring connection process. However, to cope with the increasingly complex environment of industrial robots, how to fully utilize the valuable information and improve the real-time performance of the dynamic obstacles in the environment is the focus and difficulty for path planning of industrial robots.

Finally, in terms of the collision detection process and path query, simplifying the collision detection process is the key to improving the RRT algorithm's efficiency. Specifically, it is to reduce the amount of calculation and improve the accuracy and efficiency of collision detection of the RRT algorithm. Also, the practicability and optimality of the path planning algorithm of industrial robots can be further improved by eliminating redundant nodes on the path in a more efficient way and comprehensively considering the path optimality and the kinematic and dynamic constraints of industrial robots.

## 5. The development trend of the RRT algorithm of industrial robots

Fundamentally, the ultimate goal of the design and optimization of the path planning algorithm has two aspects. One is to improve the performance of the algorithm itself, and the other is to promote the intelligent development of industrial robots by the improvement of algorithms. Therefore, this paper will summarize the future development of the RRT algorithm from two aspects:

(1) Development of the RRT algorithm.

On this aspect, the development trend of the RRT algorithm is analyzed based on the above four optimization strategies (described in Section 3.2–3.4). Then, the development trend of the RRT algorithm will be discussed in combination with other methods.

The ideal sampling process is to obtain the optimal sampling point which can not only improve the sampling efficiency but also realize the adaptability of the RRT algorithm to narrow space or maze scene. Therefore, improving the quality of sampling nodes is fundamental to solving redundant sampling and obtaining a higher quality and faster sampling process. In addition, measuring the connection process is the core of the RRT algorithm. In this process, the production of the new node can be adjusted in real-time according to dynamic obstacle information (position, velocity, and acceleration), which is the key of the RRT algorithm to realize real-time dynamic path planning (Jeong et al., 2019). However, most of the current dynamic path planning schemes using the RRT algorithm are based on re-planning (Cao et al., 2022; Lee and Song, 2023), but this re-planning method does not make full use of the information of obstacles in the environment, and its real-time performance is poor. Moreover, in practical application, the RRT algorithm has been successfully applied to many fields, such as virtual simulation (Aleotti and Caselli, 2011), artificial intelligence game development (Bauer and Popović, 2012), robotics (LaValle and Kuffner, 2001; Shkolnik et al., 2011), analog circuits (Ahmadyan et al., 2012), and protein epidemic (Vonásek and Kozlíková, 2017). Many automatic driving path planners are also inspired by the structure of the RRT algorithm (Gan et al., 2009; Li et al., 2014; Pharpatara et al., 2015). Therefore, real-time performance is the requirement of the RRT algorithm in various application scenarios. So, comprehensively considering the efficiency, real-time performance, and progressive optimality (Luna et al., 2013) is the further development of the RRT algorithm in the process of measuring connection. Furthermore, the collision detection process greatly affects the computation and security of the RRT algorithm (Liu et al., 2019). The simplified dynamic collision detection process will make the RRT algorithm more efficient and secure in the field of path planning. Finally, path planning algorithms will eventually need to be deployed into industrial robot motion controllers to control the movement of industrial robots and complete tasks. The utility of the path is particularly important. In addition to the smooth path, the attitude constraints of industrial robots should also be considered. Therefore, kinematic limitations and path quality are two important factors for industrial robots to optimize path planning algorithms.

The performance of the algorithm can be improved not only by adopting optimization strategies for itself but also by combining them with other methods. Recently, artificial intelligence technology has developed rapidly. Especially, it is more and more widely used in robotics. Particularly, deep learning algorithms and reinforcement learning algorithms have a significant effect on improving the searchability and efficiency of the RRT algorithm (Choi et al., 2021; Hao et al., 2022; Luo et al., 2022). Therefore, the combination of the RRT algorithm and artificial intelligence technology will bring a new chapter for the RRT algorithm and industrial robot intelligence. In addition, although the evolutionary algorithm is not suitable for high-dimensional space path planning of industrial robots, the fusion development of the evolutionary algorithm and RRT algorithm also improves the performance of the RRT algorithm (Montiel et al., 2015). Therefore, the combination of the evolutionary algorithm and RRT algorithm will become one of the research directions of industrial robot path planning. On the other hand, the combination of path planning algorithm and control method is also an effective way to realize path planning of industrial robots (Krämer et al., 2020; Palmieri and Scoccia, 2021). On the basis of improving the performance of the RRT algorithm, it is an effective way to realize path planning and path tracking of industrial robots by the integration of the RRT algorithm and control method. Meanwhile, more intelligent control methods (Zhao and Lv, 2023) will bring the possibility to improve the intelligence of the RRT algorithm. In addition, the heterogeneity caused by the difference in algorithm principles is the obstacle to the fusion of two different algorithms. So it is another important direction to improve the intelligence of the RRT algorithm by using advanced technology (Wang et al., 2022c) to evaluate the fusion degree of RRT algorithm and other intelligent algorithms.

(2) The aspect of improving the intelligence of industrial robots.

There are two major obstacles to the development of industrial robots: Human-robot collaboration barriers and not completely getting rid of guardrails or cages. Collaborative robots are an upgrade of industrial robots. Although it solves the two major obstacles to the development of industrial robots, it puts forward higher requirements for the safety and real-time performance of the path. The high-performance RRT path planning algorithm is not only beneficial to improve the flexibility, intelligence, and security of cooperative robots but also to expand their application fields (Krämer et al., 2020). Therefore, the optimization and improvement of the RRT algorithm are of great significance for the intelligence of industrial robots.

## 6. Conclusion

Aiming at the path planning of industrial robots, this paper comprehensively expounds on the importance of path planning of industrial robots. The characteristics of path planning of industrial robots are summarized. Then, this paper takes the RRT algorithm as the research object and investigates it for the path planning of industrial robots. Finally, with the investigation and analysis of the development and various improvement strategies, the future development directions of the RRT algorithm of industrial robots are formulated.

## Data availability statement

The raw data supporting the conclusions of this article will be made available by the authors, without undue reservation.

## Author contributions

QL planned the study design. SL conducted experiments, analyzed the data, and wrote the paper. MZ, CM, and YZ helped complete the theoretical analysis of the paper.
